# Building sustainable neuroscience capacity in Africa: the role of non-profit organisations

**DOI:** 10.1007/s11011-015-9687-8

**Published:** 2015-06-09

**Authors:** Thomas K. Karikari, Ansa E. Cobham, Iliya S. Ndams

**Affiliations:** Neuroscience, School of Life Sciences, University of Warwick, Coventry, CV4 7AL UK; Midlands Integrative Biosciences Training Partnership (MIBTP), University of Warwick, Coventry, CV4 7AL UK; Department of Anatomy, Faculty of Basic Medical Sciences, University of Calabar, Calabar, Nigeria; Department of Biological Sciences, Ahmadu Bello University, Zaria, Nigeria

**Keywords:** Africa, Neuroscience, Higher education, Non-profit organisation, Scientific capacity, Research funding

## Abstract

While advances in neuroscience are helping to improve many aspects of human life, inequalities exist in this field between Africa and more scientifically-advanced continents. Many African countries lack the infrastructure and appropriately-trained scientists for neuroscience education and research. Addressing these challenges would require the development of innovative approaches to help improve scientific competence for neuroscience across the continent. In recent years, science-based non-profit organisations (NPOs) have been supporting the African neuroscience community to build state-of-the-art scientific capacity for sustainable education and research. Some of these contributions have included: the establishment of training courses and workshops to introduce African scientists to powerful-yet-cost-effective experimental model systems; research infrastructural support and assistance to establish research institutes. Other contributions have come in the form of the promotion of scientific networking, public engagement and advocacy for improved neuroscience funding. Here, we discuss the contributions of NPOs to the development of neuroscience in Africa.

## Introduction

The centralised role of the nervous system in coordinating both conscious and unconscious bodily processes, actions and perceptions makes neuroscience an important discipline in biomedical research (Lewis 2006). Yet, advances in neuroscience are not limited to biomedical applications, but are extended to other areas such as education, psychology, sociology, economics and criminal justice. It is therefore not surprising that many interdisciplinary neuroscience subspecialties have been developed in recent years; these include neuroeconomics, educational neuroscience, neurolaw, neuropsychology, neuroleadership and neurosociology (Camerer et al. 2005; Rock and Schwartz 2006; Hruby 2012; Takahashi 2012; Franks and Turner 2012; Andrewes 2013). A general concept that drives neuroscience research is that improved understanding of the development, anatomy and physiology of the nervous system and how the nervous system communicates with other bodily structures would help to elucidate the biological basis of behaviour, health and disease (Lewis 2006). With this motivation, scientists have conducted research to obtain further insights into the neural basis of organismal development, behaviour and disease, and are applying the new knowledge gained to, for example, design more effective treatments for neurological, neurodevelopmental and neurobehavioural disorders (Srikanth and Kessler 2012; Gendelman et al. 2015).

While these developments in neuroscience provide hope to improve our understanding of the biological basis of organismal health and development, large proportions of these advances have come from research laboratories in continents that are more developed than Africa (Adams et al. 2010; Yusuf et al. 2014). A plausible explanation for this would be that there exists a disparity in the quality and quantity of neuroscience research between Africa and more scientifically-developed continents such as Europe and North America (Yusuf et al. 2014). Indeed, many African countries lack fundamental competences in neuroscience and related disciplines, resulting from challenges such as the chronic lack of teaching and research infrastructure, research funding and expert instructors (Juliano 2012; Sofola 2014). Moreover, world-class neuroscience training programmes are lacking in the continent, meaning that talented students have limited local avenues to be trained in neuroscience (Juliano 2012; Karikari et al. 2015). Aside from these, some of the African students sponsored for higher education abroad do not return home upon completing their studies (Docquier and Rapoport 2011). In effect, world-class neuroscience research conducted locally in Africa is deficient (Juliano 2012). For this reason, African countries usually rank low in global research productivity scores. For example, a Global Research Report published by the Thomson Reuters Corporation showed that South Africa, Egypt, Tunisia, Morrocco and Nigeria are the leaders in neuroscience and behaviour research in Africa, yet the combined output of these five countries accounts for only 0.36 % of the global output in this research area (Adams et al. 2010). With the burden of neurological diseases in Africa predicted to increase in the near future, the serious economic, health and social burdens associated with these diseases might also increase (Menken et al. 2000; Dua et al. 2006; George-Carey et al. 2012). There is therefore an urgent need for long-term planning and support for neuroscience education and research in Africa, to extend our understanding of disease mechanisms and help develop more effective diagnostic, treatment and care platforms.

Over the years, several approaches have been employed to improve neuroscience research in Africa. They include: (1) sponsoring promising African researchers for short-term training in leading universities abroad, and (2) awarding scholarships for graduate training abroad (Yusuf et al. 2014). The rationale behind these approaches was that the beneficiary scientists would benefit from high-class research training in the host institutions, which they would bring to support local research activities in their home institutions in Africa once they return. Notwithstanding the apparent benefits of these approaches, they have their downsides. For example, only a few African neuroscientists get selected for prestigious fellowships like the neuroscience summer school fellowships awarded by the US-Canada Regional Office of the International Brain Research Organization (IBRO). Moreover, as some of the beneficiaries of sponsored graduate training abroad do not return to work in Africa after their training, Africa loses some of her elite scientists to other continents. Due to the aforementioned reasons, the existing approach of training African scientists abroad should be supplemented with other initiatives that will be more inclusive, cost-effective and more sustainable in the long term. One of these much-needed plans is to focus on providing world-class neuroscience training for African neuroscientists locally, without having to send these trainees abroad. Institutions such as IBRO, Society of Neuroscientists of Africa (SONA) and specific national neuroscience societies have been instrumental in providing this kind of training (Yusuf et al. 2014).

To ensure that more scientists in Africa benefit from outstanding neuroscience training and are also supported to conduct high-impact research, some science-based non-profit organisations (NPOs) have been partnering the African neuroscience community to provide modern research infrastructure and training courses conducted at African universities. Other contributions have come in the form of promoting pan-African scientific networking, public engagement and advocacy for improved neuroscience funding. In this article, we discuss the contributions of these NPOs to neuroscience capacity building in Africa. This discussion will focus on NPOs that have provided support specifically for neuroscience education and research within Africa. The NPOs, identified through their contributions to the specific areas of capacity building discussed later in the article, include; Teaching and Research in Natural Sciences for Development in Africa (TReND in Africa; www.trendinafrica.org), Korle Bu Neuroscience Foundation (KBNF; http://kbnf.org), NeuroLeadership Institute (https://www.neuroleadership.com), The Dana Alliance for Brain Initiatives (http://www.dana.org) and Seeding Labs (http://seedinglabs.org). We will also highlight how some of these NPOs have partnered with scientific societies, funding agencies and universities in the delivery of their support initiatives, and how other organisations and individuals can emulate such strategies.

## Neuroscience training programmes

Neuroscience research output in Africa is low when compared to what exists in more developed continents (Adams et al. 2010). While Africa recently recorded improvements in the rating of locally-conducted scientific research, the continent’s contribution to global scientific research remains low (The World Bank 2014). For example, in 2008, Africa contributed only 2.0 % to the global collection of scientific publications (UNESCO 2010). Also, sub-Saharan Africa contributed only 0.72 % to the global research output in 2012 (The World Bank 2014). One of the major reasons accounting for this low research productivity is the lack of topnotch training programmes and curricula especially those with a focus on hands-on, laboratory research-intensive programmes (Juliano 2012).

Sustainable improvements in neuroscience education and research in Africa would require developing the expertise of local scientists to international standards, to enable them conduct high-quality research and to impart the requisite knowledge to the next generation of neuroscientists (Yusuf et al. 2014; Muindi and Keller 2015). For this reason, various NPOs have organised training workshops, have supported the establishment of and degree programmes in neuroscience for African scientists.

One of the leading NPOs in organising neuroscience workshops for African scientists is TReND in Africa. With funding from IBRO, The Company of Biologists and other agencies, TReND in Africa has been organising neuroscience summer schools for African scientists since the year 2011 (Muindi and Keller 2015; Table 1). To date, about 100 scientists from over a dozen African countries have benefitted from these courses. Focused primarily on introducing scientists to the use of invertebrate organisms (such as the fruit fly, *Drosophila melanogaster*) as powerful-yet-cost-effective model organisms for neuroscience research, the annual neuroscience summer courses also provide training in the development of experimental techniques for cutting-edge research (Parslow 2014; Dunne 2014). Participants are also trained in how to build their own labware using open source resources (Baden et al. 2015). By providing scientists with inexpensive means of conducting leading research, this course has been helping beneficiaries to rise above the lack of research tools and materials in some African laboratories, by encouraging creativity and a “do-it-yourself” spirit among scientists (Dunne 2014). Additionally, TReND in Africa offers training programmes in next generation sequencing data analysis to enable more African scientists participate in genomics research particularly neurogenomics (Karikari and Aleksic 2015). Many beneficiaries of the TReND in Africa neuroscience schools have gone ahead to make impacts in their research and education. For example, some of the alumni recently applied their knowledge in the assembly and use of an electrophysiology kit gained during the neuroscience workshops to study the effect of cyanide-induced oxidative stress on mammalian cerebral physiology (Ogundele et al. 2014). On the corporate front, the University of Stellenbosch Business School in South Africa has partnered with the NPO NeuroLeadership Institute to introduce a certificate programme in neuroleadership (USB-ED 2012; Table 1). Originally coined by (Rock and Schwartz 2006), the term “neuroleadership” refers to the aplication of neuroscience advances to help make organisational transformation succeed. Neuroleadership helps corporate organisations to maximise their potentials by utilising neuroscience breakthroughs about how the human brain functions to guide organisational decisions (USB-ED 2012). It is inspirational to note that business professionals are being trained to apply neuroscience concepts in the African business environment. This development may be an indication of future partnerships between neuroscientists and business organisations in Africa, towards applying neuroscience knowledge to promote industrial productivity.

**Table 1:** Summary of activities led by non-profit organisations that are helping to build capacity for neuroscience in Africa

Area	Support programmes	Examples	References
Training	Online training and open source resources	TReND in Africa’s open neuroscience project and *Build your own lab equipment* workshops.	(http://openeuroscience.com; http://trendinafrica.org/activities/open-source/; Baden et al. 2015)
	Short courses and workshops	TReND in Africa’s courses in: neurogenetics, genomics, bioinformatics, molecular biology and building open source tools. NeuroLeadership and the University of Stellenbosch in South Africa have started a certificate course in neuroleadership.	(USB-ED 2012; Parslow 2014; Yusuf et al. 2014; Dunne 2014; Karikari and Aleksic 2015; Muindi and Keller 2015)
	University degree programmes	The Korle Bu Neuroscience Foundation is helping to establish a graduate neuroscience programme at the University of Ghana in Accra. TReND in Africa is supporting the establishment of a graduate neuroscience programme at Kampala International University, Uganda.	(Parslow 2014; Yusuf et al. 2014; Dunne 2014; KBNF 2015)
Building infrastruc tural capacity	Research infrastructure support	KBNF has helped universities and hospitals in Ghana and Nigeria to acquire clinical neuroscience infrastructure. TReND in Africa has supported universities in Uganda, Tanzania and Nigeria to obtain much-needed research equipment. Seeding Labs has supported several African institutions with laboratory equipment to support research activities.	(Yusuf et al. 2014; KBNF 2015; Muindi and Keller 2015; http://seedinglabs.org).
Public engagement about neuroscience		Volunteers of TReND in Africa and KBNF have engaged African students, community groups and policy makers about neuroscience.	(Dunne 2014; KBNF 2015; Karikari et al. 2015; Muindi and Keller 2015)

Top-quality neuroscience degree programmes offered in African universities are required to provide learners with in-depth training in the field (Yusuf et al. 2014). Such programmes would help to train scientists in the requisite knowledge and skills to conduct contemporary neuroscience research. Yet, neuroscience degree programmes are lacking in many parts of the continent (Juliano 2012; Yusuf et al. 2014). For example, Ghana currently has no degree programme in neuroscience, thus making it difficult for Ghanaian students scientists and clinicians to obtain advanced training in this field (Karikari et al. 2015). In order to overcome this, KBNF, which is an NPO dedicated to supporting clinical neuroscience education, research and medical care, is supporting the University of Ghana Medical School to establish Ghana’s first-ever neuroscience graduate programme (KBNF 2015; Table 1). By partnering with the Vancouver General Hospital in Canada, KBNF is helping to train African physicians in neuropathology until the proposed neuroscience programme in Ghana becomes operational (KBNF 2015). Similarly, TReND in Africa has started a partnership with the Kampala International University (KIU), Uganda, to establish an interdisciplinary neuroscience graduate programme for the East African community (Yusuf et al. 2014; Table 1). Through such training initiatives, these NPOs are making invaluable contributions to building the long-term sustainability of neuroscience in Africa by ensuring that African students obtain quality training in their home continent, thereby cutting training costs and consequently fighting against brain drain.

## Research infrastructure

To promote neuroscience research in African universities, equitable investments in building the needed infrastructural capacity to enable researchers conduct groundbreaking research are essential (Yusuf et al. 2014; Muindi and Keller 2015). Provision of these needed research facilities would help African scientists to accelerate their efforts aimed at finding solutions to neuroscience-related challenges that affect the African society; such as developing effective treatments for tropical diseases that affect the brain and spinal cord (Yusuf et al. 2014; KBNF 2015).

Some NPOs operating in Africa have been instrumental in providing the needed equipment support to boost local neuroscience research. For example, TReND in Africa has developed a clever strategy of supporting African institutions with functional research tools. To request for equipment support, leaders at African insitutions file application via the organisation’s website (http://trendinafrica.org), listing the equipment they need and the contact person for correspondence. TReND in Africa volunteers then appeal to leading institutions in Europe and North America which might have these equipment as surplus-yet-functional resources to donate. Upon donation, the equipment are packed and shipped to the African partner university; the receiving institution covers only the shipping cost. This approach provides a cost-effective means for African institutions to obtain research equipment. To date, KIU in Uganda has been the largest beneficiary of this support system, leading to the establishment of an Institute for Biomedical Research (Yusuf et al. 2014; Table 1). Other universities, such as Gombe State University and University of Maiduguri, both in Nigeria, as well as the Dar es Salaam campus of KIU have also benefitted. Using a similar approach, KBNF has supplied the Korle Bu and University of Benin teaching hospitals in Ghana and Nigeria respectively with hospital equipment and supplies to support clinical neuroscience training and research (KBNF 2015; Table 1). The organisation is also supporting the establishment of a West African neuroscience research department at the University of Ghana (KBNF 2015). Seeding Labs is another organisation that has provided research infrastructural support to African institutions to help reduce the expense involved in conducting world-class research. The organisation has provided laboratory equipment and materials worth several hundreds of thousands of dollars to research institutions in Africa. This support system has contributed to advancing research and educational activities in several countries (Zagorski 2011; Yusuf et al. 2014). In addition to the provision of equipment, Seeding Labs has partnered with the Novartis Institute of Biomedical Research (NIBR) to implement a summer fellowship programme through which promising early-career scientists from Africa are trained at NIBR laboratories in the United States (Zagorski 2011).

## Free and open-source educational resources

A potentially cost-effective approach to upgrading science, and for that matter neuroscience, in Africa would be to adopt free and open source educational resources (Yusuf et al. 2014; Karikari 2015a; Baden et al. 2015). The several free, publicly-accessible, open source resources (FOSR) in neuroscience provide scientists with unrestricted access to research tools and materials. FOSR also allow citizen scientists and pre-university students to explore their interests in neuroscience.

To promote the use of FOSR, especially in resource-limited research environments in Africa, TReND in Africa’s training programmes dedicate special attention to providing trainees with hands-on guidance to develop their own laboratory equipment, while also guiding learners through how to use already-available open source tools (Yusuf et al. 2014; Baden et al. 2015). A notable example is that participants at the organisation’s neuroscience summer courses are trained in how to assemble and use simple, low-cost electrophysiology kits such as the Spiker box from Backyard Brains (Marzullo and Gage 2012; Yusuf et al. 2014). Participants use their assembled kits for experimentation during the course, and subsequently keep these kits to support research and training in their home countries. This way, the organisation is helping to instill a culture of inventiveness and improvisation among African neuroscientists and help them rise above the usual excuse of lack of resources. To make FOSR in neuroscience more accessible to everyone, volunteers of TReND in Africa have established the open neuroscience project (http://openeuroscience.com; Table 1) to share information about available neuroscience-related open source tools. Also, the NPO is making preparations to hold its first open source workshop to train African scientists in how to build their own research tools using low-budget materials (Table 1).

## Scientific networking and volunteering

A well-discussed challenge of scientific research in Africa is the lack of intra-continental research collaborations (The World Bank 2014; Karikari 2015a). African scientists usually look externally, instead of turning to their African colleagues, for research collaborators (The World Bank 2014). This lack of intra-continental collaboration has had negative tolls on the continent’s scientific output and independence (The World Bank 2014). To be able to set and direct joint research agenda geared towards addressing the continent’s debilitating challenges, platforms should be provided for African scientists to meet, interact and potentially collaborate (Chu et al. 2014).

Through their activities, NPOs such as TReND in Africa have provided opportunities for African scientists to network. For example, participants with varying educational backgrounds and academic expertise from different African countries are selected onto the courses. The organisation’s recent neuroscience course held in August 2014, for instance, welcomed seventeen participants from ten African countries (Parslow 2014). At the end of each course, participants share their contact information in order to stay in touch; this contact list is cummulatively updated after subsequent courses. Course faculty usually include resident scientists in Africa as well as their colleagues from abroad (Parslow 2014). This approach provides many opportunities for potentially fruitful interactions among course attendees. These include the following: (1) Alumni can collaborate to design and conduct joint research projects, taking advantage of their varying expertise, backgrounds and interests. For instance, a four-member team comprising a computational neuroscientist, an anatomist, a physiologist and a biochemist can jointly design and execute a neuroscience project. (2) The programme allows African graduate students and early-career scientists to meet and interact with their senior colleagues. These junior scientists can benefit from career guidance, mentoring and other forms of assistance from their senior fellows working in different institutions and countries. (3) Senior scientists can also be trained in contemporary research techniques (for example, in computational neuroscience) from their younger colleagues who may be better versed in these techniques. (4) The participants as well as the African faculty who teach on the courses could potentially develop partnerships among themselves and also with the non-African faculty members. Although it may be too early to comment on the impact on this approach by TReND in Africa, initial results do suggest that it will be a viable means to promote pan-African research.

## Public engagement about neuroscience

For neuroscience to become a more recognised scientific discipline among the African public, neuroscience tools and concepts should be made more accessible and understandable to non-scientists. This is because neuroscience advances are strongly correlated with economic and social development (Camerer et al. 2005; Blakemore 2008), and should therefore be made accessible to everyone. The more the African public has access to neuroscience information and understand this information, the more likely is it that neuroscience will be better adopted to improve our everyday lives. Moreover, due to the difficulty in teaching neuroscience to students both at the higher education and pre-university levels, African neuroscientists can support school teachers and colleague faculty members to integrate innovative teaching approaches (such as computational techniques) that support inquiry-based learning (Karikari 2015b, c).

In order to improve the public understanding of neuroscience in Africa, TReND in Africa volunteers have reached out to over 1000 people across Africa through outreach activities in countries such as Ghana, Uganda, Nigeria and Tanzania (Parslow 2014; Dunne 2014; Karikari et al. 2015; Muindi and Keller 2015). For example, as part of the Brain Awareness campaigns designed to promote societal awareness of brain health and diseases, these volunteers develop outreach activities targeted at pre-university students. Since neuroscience has not been sufficiently integrated into the school curricula in Africa, the primary goals of these school outreach activities have been to motivate learners in their studies, increase their understanding of specific scientific topics, and to introduce them to exciting scientific careers in neuroscience (Karikari et al. 2015). These events have been supported with educational resources kindly donated by The Dana Alliance for Brain Initiatives. Moreover, to argue for the need to improve investments in tertiary-level science education in Africa, TReND in Africa volunteers have been involved in advocacy efforts aimed at convincing African governments to increase support for scientific research. For instance, the organisation had representation at IBRO’s Global Advocacy Symposium 2014 to argue for the need for improved governmental contribution to local research (Parslow 2014). Aside from TReND, another organisation supporting public education in neuroscience in Africa is KBNF. KBNF members do organise public outreach activities to empower the African public against preventable neurological disorders, such as spina bifida (KBNF 2015).

Through the aforementioned public engagement activities, the NPOs are helping to increase interaction between African neuroscientists and the public in order to promote literacy in neuroscience and increase the public’s interests in, and attitudes towards, the discipline. Also, by organising activities to introduce pre-university students in Africa to careers in neuroscience and related disciplines, these NPOs are guiding the continent’s next generation of leaders to make informed career choices, to ensure that the talents of these future professionals are put to the best use.

## Conclusion

NPOs are becoming an excellent source of support for neuroscience development in Africa. These organisations have helped to train African scientists in contemporary techniques for neuroscience research and education, and have also contributed to the establishment of research institutes and the provision of research infrastructure. These indicate that building bridges in neuroscience (between African scientists, and also between scientists in Africa and their colleagues abroad) is one of the effective means of building neuroscience capacity in the continent. Learning from this, neuroscientists in Africa (and scientists in other disciplines) can devise similar strategies to develop mechanisms of overcoming the chronic challenges that have caused science in the continent to lag behind other parts of the world. This way, resident scientists in Africa would obtain improved access to urgently-needed tools and materials in order to promote their teaching and research activities. We also call on African governments, industries and other NPOs to increase investments in neuroscience in order to help advance healthcare, education, business, economics and social wellbeing in the continent.

IBRO, International Brain Research Organization; KBNF, Korle Bu Neuroscience Foundation; KIU, Kampala International University; TReND in Africa, Teaching and Research in Natural Sciences for Development in Africa; NPO, non-profit organisation
